# Prescription, Dispensation, and Generic Medicine Replacement Ratios: Influence on Japanese Medicine Costs

**DOI:** 10.5539/gjhs.v8n1p29

**Published:** 2015-05-15

**Authors:** Masayuki Yokoi, Takao Tashiro

**Affiliations:** 1 The School of Graduate Studies, The Open University of Japan, Chiba, Japan; 2 Pascal Pharmacy, Chiba, Japan

**Keywords:** costs, generic, medicine, multiple, reducing, regression, separation, system

## Abstract

This study used publicly available data to examine the effect of the separation of dispensing and prescribing medicines between pharmacists in pharmacies and doctors in medical institutions (the separation system) and the generic medicine replacement ratio on the cost of various medicines in Japanese prefectures. For Japanese medical institutions, participation in the separation system is optional. Consequently, the expansion rate of the separation system for each administrative district is highly variable. In our multiple regression analysis, the dependent variables were the costs of *daily medicines*, specifically, *total*, *internal*, *external*, and *injection medicines*, as well as *medical devices*, and the independent variables were the expansion rate of the separation system and generic medicine replacement ratio. The expansion rate of the separation system showed a significant negative partial correlation with the daily costs of *total*, *internal*, and *injection medicines* as well as *medical devices*. Moreover, the rate of replacing brand name medicines with generic medicines showed a significant negative partial correlation with the daily costs of *total* and *internal medicines*. However, *external* and *injection medicines* and *medical devices* did not because only a few or no generic products of these types were sold in the Japanese market. Otherwise, expansion of the separation system was effective in reducing medicine costs, except in the case of *external medicines*. This suggests that the cost efficiency effect of the separation system does not function all the time.

## 1. Introduction

There are several studies that examine the effectiveness of interventions in clinical care by pharmacists. For example, Rollason and Voqt (2003) reported the effectiveness of interventions by pharmacists in reducing polypharmacy in a systematic review; Saokaew et al. (2013) showed that pharmacists participating in warfarin therapy management is a cost-effective intervention; and Touchette et al. (2014) discussed economic evaluations of clinical pharmacy services.

With regard to pharmacists’ cost-effective activities, several studies have examined the influence of the separation of dispensing and prescribing medicines on medicine costs between pharmacists in pharmacies and doctors in medical institutions. This system (hereafter, the separation system) has been introduced and become familiar over several hundred years in European, North American, and Middle Eastern countries. On the other hand, Asian countries, for example the Republic of Korea and Japan, only began the widespread introduction of the separation system in the latter half of 20^th^ century, while in China, the government is still hesitating to introduce the system.

Japan began the widespread adoption of the separation system at the end of the 20^th^ century because the government expected it would reduce medicine costs, especially medicine costs, by decreasing the number of duplicate prescriptions and reducing the economic motive for prescriptions. However, little evidence has been reported to support these expectations. It has been pointed out that most economic evaluations of clinical pharmacy intervention suffer from several methodological limitations relating to the absence of a control group without clinical pharmacy interventions (De Rijdt, Willems, & Simoen, 2008). In addition, the economic effects of the separation system have not yet been studied quantitatively because it is difficult to precisely compare the objects under study.

Therefore, it has been unclear whether the system promotes the proper prescription of medicines or reduces medicine costs (Nakamura, Nagaki, Iizuka, & Fujii, 1989; Watanabe, 1996), although more recently, Yokoi and Tashiro (2014) examined the influence of the separation system on medicine costs in Japan. Our multiple regression analysis revealed that the expansion rate of the separation system and the rate of replacing brand name medicines with generic medicines had a significant negative partial correlation with daily internal medicine costs. Thus, the separation system was as effective in reducing internal medicine costs as the use of generic medicines and it remains unclear how the separation system influences the costs of total, injection, and external medicines or the costs of medical devices, for which Japanese pharmaceutical laws allow prescriptions to be written for.

To address this, the present study hypothesized that the separation system has contributed to reduced daily total medicine costs. Moreover, we attempted to verify the specific contribution of the separation system to reduce various medicine costs in Japan and under what circumstances it was functional.

## 2. Method

Japan has a universal national health insurance system and all Japanese people belong to a specific public health insurance system. Japan’s system is one of universal care and, with some exceptions, such as welfare for livelihood protection, insurance is divided into social health insurance for full-time employees and National Health Insurance for the others, such as students and the self-employed. Therefore, by collating data from both insurance types, we can cover Japan’s total data for medical insurance.

We can estimate the health and medicine costs of the whole nation. Importantly, because of frequent changes in the Japanese insurance system, it is difficult to evaluate the exact effect of each factor on medicine costs. We can clarify this by analyzing contemporary national data.

In the present study, we examined the financial efficiency of the separation system by evaluating data from each of Japan’s prefectures. Japanese prefectures are broad-based common municipal corporations that divide Japan into 47 administration districts. Using the public health insurance database of the Japanese Ministry of Health, Labour and Welfare (MHLW), we calculated daily costs of internal, external, and injection medicines as well as medical devices, like injection needles for patients’ own usage as well as those for total costs. The results were expected to provide a clear understanding of the cost efficiency of the separation system for various medicines and medical devices. We performed multiple linear regression analysis as follows: the dependent variables were daily medicine costs for internal medicines, external medicines, injection medicines, and medical devices per prescription. The independent variables were the expansion rate (%) of the separation system and the base ratio (%) of brand-name medicines replaced with generic medicines.

### 2.1 Dependent Variables

We obtained 2013 fiscal data on average medicine cost and dosage days per prescription for each prefecture (Ministry of Health, Labour and Welfare [MHLW], 2013). We then divided the average medicine charge per prescription by the dosage days per prescription to obtain the daily medicine cost.

Nationwide data on prescriptions are available from insurance applications for medicine expenses received from pharmacies, 99.9% of which are covered currently by medical insurance in Japan. Table 1 shows the descriptive statistics of the dependent variables used in the analysis of the data from the 47 Japanese prefectures. The definition of each item is as follows. Internal medicines are to be taken orally and usage dose is decided on prescription. External medicines are patches, enemas, suppositories, eye drops, nasal drops, ear drops, and gargles. Injection medicines are only for outpatient use at home, for example, insulin, hormones, and anticancer treatments. Medical devices include injection needles and catheters for outpatient use at home. We analyzed the daily costs of these items as dependent variables and performed multiple linear regression analysis on these five items as dependent variables.

**Table 1:** The statistical data of objective variables, daily cost (US cent, 1 cent=1 yen)

Dependent variable	Total	Internal medicines	External medicines	Injection medicines	Medical devices
Data Number	47	47	47	47	47
Mean	306.6	256.4	36.05	11.316	0.7384
Max	346.8	295.7	40.48	16.595	1.3869
Min	275.2	229.4	30.06	8.197	0.3311
S.D.	17.5	15.7	2.4	1.985	0.2327

Max=maximum, Min=minimum, S.D.=standard deviation.

In Table 1, the number of observations equals the number of Japanese prefectures. Each cost is per prescription.

### 2.2 Independent Variables

The independent variables were the expansion rate (%) of the separation system and the base ratio (%) of brand-name medicines replaced with generic medicines. We expected these variables to have a considerable influence on daily medicine costs (Yokoi & Tashiro, 2014). In addition, the reason we selected these two factors was as follows. It is well known that using generic medicines is cost effective. Therefore, we tried to compare the cost effectiveness of the separation system with generic medicines.

#### 2.2.1 Expansion Rate of the Separation System

We obtained 2013 fiscal data of the expansion rate (%) of the separation system among administrative divisions in Japan (Japan Pharmaceutical Association [JPA], 2013). The expansion rate of the separation system estimated by the JPA is calculated according to statistics provided by the annual reports of the Insurance Association for Private Organization and National Health Insurance. An estimation level of the overall prescription number of sheets that should be prescribed in Japan was calculated by the JPA from the ratio of medical insurance receipts using medicine for medical treatment days from statistics for the past five years. As a result, it was estimated that 10.6% of medical treatment days was used for medicines in dental practice and 67.1% for medicines in medical practices, except dental practices. Therefore, the overall number of prescription sheets that Japan should be prescribing was calculated at 10.6% of the medical treatment days in dental practice and 67.1% of those in medical practice. The expansion rate of the separation system of each Japanese prefecture is calculated by the number of published prescription sheets that can be found in both previous insurance data divided by the estimated overall number of prescription sheets. The JPA has publicized the expansion rate of the separation system of each Japanese prefecture on their website. In Japan, these data are recognized as data of the formal expansion rate of the separation system. We used this data for our multiple linear regression analysis as independent variables.

#### 2.2.2 Generic Medicine Replacement Ratio

These data were taken from the MHLW website—specifically, they were based on trends in the composition of medical expenses for computation processing in 2013 (MHLW, 2013). From these data, we calculated the rate of brand name medicines replaced with generic medicines (hereafter, the generic medicine replacement ratio) for each prefecture. These data are precise because they are based on data for universal health care and medical insurance, which covers 99.9% of Japanese medical care.

### 2.3 Data Analysis

Multiple regression analysis was conducted using the various daily medicine costs per prescription as the dependent variable and the spreading rate and generic medicine replacement ratio as independent variables. All statistical analyses were performed using Excel Statistics 2012 (Society Information Service Co., Japan).

## 3. Results

The results show Tables 2–7. Table 2 shows the results of multiple correlation analysis for each daily medicine cost. This table designates each multiple correlation coefficient R and P-value.

**Table 2:** Multiple correlation coefficient and P-value

Dependent variable	Total	Internal medicines	External medicines	Injection medicines	Medical devices
Multiple correlation coefficient R	0.681	0.671	0.219	0.449	0.332
P-value	<0.0001	<0.0001	0.338	<0.01	0.07

**Table 3:** Coefficient of multiple regression analysis (dependent variable: *Total*)

Independent Variable	Expansion rate (%)	Generic medicine replacement (%)	Constant
Standard regression coefficient	-0.430	-0.469	-
Partial correlation coefficient	-0.502	-0.535	-
P-value (partial correlation)	<0.001	<0.0001	<0.0001

Y_1_= -0.765X_1_ -2.082X_2_ +456.965

Y_1_: *Total medicine cost,* X_1_: *Expansion rate* (%), X_2_: *Generic medicine replacement* (%).

**Table 4:** Coefficient of multiple regression analysis (dependent variable: *internal medicine*)

Independent Variable	Expansion rate (%)	Generic medicine replacement (%)	Constant
Standard regression coefficient	-0.400	-0.483	-
Partial correlation coefficient	-0.471	-0.542	-
P-value (partial correlation)	<0.001	<0.0001	<0.0001

Y_2_= -0.644X_1_ -1.938X_2_ +392.513

Y_2_: *Internal medicine cost*, X_1_: *Expansion rate (%)*, X_2_: *Generic medicine replacement (%).*

**Table 5:** Coefficient of multiple regression analysis (dependent variable: *external medicine*)

Independent Variable	Expansion rate (%)	Generic medicine replacement (%)	Constant
Standard regression coefficient	-0.032	-0.212	-
Partial correlation coefficient	-0.034	-0.210	-
P-value (partial correlation)	0.823	0.160	<0.0001

Y_3_= -0.008X_1_ -0.057X_2_+6.32

Y_3_: *External medicine cost*, X_1_: *Expansion rate (%)*, X_2_: *Generic medicine replacement (%).*

**Table 6:** Coefficient of multiple regression analysis (dependent variable: *injection medicine*)

Independent Variable	Expansion rate (%)	Generic medicine replacement (%)	Constant
Standard regression coefficient	-0.453	0.085	-
Partial correlation coefficient	-0.448	0.094	-
P-value (partial correlation)	<0.01	0.535	<0.0001

Y_4_= -0.092X_1_ -0.043X_2_ +15.24

Y_4_: *Injection medicine cost*, X_1_: *Expansion rate (%)*, X_2_: *Generic medicine replacement (%).*

**Table 7:** Coefficient of multiple regression analysis (dependent variable: *medical device*)

Independent Variable	Expansion rate (%)	Generic medicine replacement (%)	Constant
Standard regression coefficient	-0.336	0.055	-
Partial correlation coefficient	-0.332	0.058	-
P-value (partial correlation)	<0.05	0.702	<0.05

Y_5_= -0.008X_1_ +0.0033X_2_ +1.098

Y_5_: *Medical device cost*, X_1_: *Expansion rate (%)*, X_2_: *Generic medicine replacement (%).*

As for partial correlations, Tables 3–7 show the results of the relationships between each dependent variable and the independent variables. The tables show each standard regression coefficient, partial correlation coefficient, and P-value (partial correlation). These coefficients partially designate the relationships between each dependent variable and the expansion rate or generic medicine replacement rate. In addition, each multiple regression function is designated under each table. Each P-value is for partial correlation with each dependent variable, namely, *total, internal, external*, and *injection medicine* costs as well as *medical device* costs.

The results reveal a significant partial correlation for the expansion rate and the generic medicine replacement ratio for the items included in the analysis of the costs of the *total* and *internal medicines*. In addition, for the cost of *injection medicines* and *medical devices*, a significant partial correlation was observed only in the expansion rate and not in the generic medicine replacement ratio. For the cost of *external medicines*, we observed no significant partial correlation for either the expansion rate or the generic medicine replacement ratio.

## 4. Discussion

We found significant multiple correlation, except for the costs of *eternal medicines* and *medical devices*. Therefore, the costs of *total, internal*, and *injection medicines* are considered to be influenced by the expansion rate of the separation system and the generic medicine replacement ratio.

With regard to partial correlation, we found that the daily costs of *total* and *internal medicines* had significant partial correlations with two factors—the expansion rate and the generic medicine replacement ratio. The marketing share of the costs of *internal medicines* is 84% of *total medicine* costs in Japan. Therefore, *total* costs were influenced easily by the costs of *internal medicines*. In addition, the costs of *injection medicines* and *medical devices* had significant partial correlations with the separation system. First, this study revealed that the separation system had a strong influence on reducing costs for *total, internal*, and *injection medicines* as well as *medical devices* (we note that *internal medicine* costs are revealed in Yokoi & Tashiro, 2014).

Using generic medicines to reduce medicine costs is a well-known practice. This is an independent factor from the effect of the expansion of the separation system. In our study, the magnitude of these partial correlation coefficients for the generic medicine replacement ratio was similar to that of the expansion rate of the separation system in *total* and *internal medicines*. Thus, with regard to reducing *total* and *internal medicine* costs in Japan, the separation system is as important as the use of generic medicines.

Otherwise, as for the *injection medicine* and *medical device* costs, the absolute value of the partial correlation coefficient for the separation system is more than the generic medicine replacement ratio. In addition, only the separation system has a significant correlation with these two costs; the generic medicine replacement ratio does not. This means that the separation system influences these two costs.

There are very little data on the constant effect of the separation system on medicine cost reduction. However, we were able to identify a fixed effect because the separation system is not mandatory in Japan; instead, prescribing doctors in medical institutions can choose whether to participate. Consequently, we were able to calculate the daily medicine cost data under various expansion rates and the effect of the separation system on medicine costs. Although this study did not determine why these medicine costs decreased strictly with the increased expansion rate, it nonetheless provided valuable evidence for the economic efficiency of the separation system.

### 4.1 External Medicine Costs

We could observe neither a statistically significant negative multiple correlation nor a partial correlation between the *external medicine* costs and the two independent factors, the expansion rate and the generic medicine replacement ratio.

The annual sales of Japanese external medicines for prescription are about US# 2.4 billion. Of this, the sales share of patch-type light pain-killer medicines for waist, shoulder, elbow, knee, and joint pains, such as nonsteroidal anti-inflammatory drugs, is about 70%. Therefore, this result is considered to be significantly influenced by these sales. Patch-type light pain-killer medicines are the most popular external medicines and these are covered by insurance in Japan. As for the generic medicine replacement ratio, Japanese patients worry about the feeling and stickiness of patch-type medicines as most such generic medicines are worse than brand-name medicines. Therefore, generic patch-type medicines are not very popular in the Japanese market.

External medicines, like light pain-killer patches, are widely adopted by public insurance in Japan. Outpatients can purchase them for cheap prices using insurance. Thus, outpatients prefer to have external medicines prescribed at medical institutions than purchasing such medicines at drug stores, where medicines for sale are not covered by public insurance. Under these circumstances, Japanese outpatients habitually intervene to have external medicines prescribed; for example, they request doctors or pharmacists to prescribe or dispense much more than necessary. They often request such medicines to keep as their own stock or for their acquaintances’ usage. The pharmaceutical efficiency of light pain-killer patches are considered weak. Therefore, these prescriptions are easily influenced by patients’ disposition toward such medicines.

Patients must purchase medicines that have been prescribed by doctors as they cannot decide themselves what to purchase. If a doctor also sells medicine, then the person who decides what to prescribe is also a seller. The separation system is intended to settle this contradiction by ensuring that prescribing doctors decide what patients buy but not sell the product; on the other hand, pharmacists do not decide what patients buy but they sell and dispense the products.

In the medicine market, buyers, namely, patients, cannot judge which medicines to buy because of lack of medical knowledge. Therefore, when doctors or medical institutions sell medicines, they also decide what patients should buy. The reason for the division of prescribing and dispensing is to avoid the purchase of medicines by patients arising out of the circumstances of the seller.

However, in some cases, patients can decide to buy patch-type light pain-killer medicines, illustrating that the mechanism of the separation system does not function well. By way of evidence, most patch-type light pain-killer medicines contain medicated ingredients that are the same as those of prescribed medicines sold at pharmacies in Japan; patients can buy them using their own discretion and do not need prescriptions or doctors’ advice at drugstores. Patients do not need to change their actions to purchase patch-type pain-killer medicines. The market situation of *external medicines* is similar to that of the drugstore market in terms of self-regulation.

It was reported in the press recently (yakuji-nippo 2015) that the Japanese government has begun to consider whether to remove *external medicines*, such as poultice included light pain-killer patches, from public insurance. Thus, the economic-efficiency effect for the separation system could be weak owing to patients’ disposition.

### 4.2 Injection Medicine Costs

The costs of *injection medicines* had a significant partial correlation with the separation system, which is the same result as that for *internal* and *total medicines*. The economic efficiency of the separation system is considered to work in the field of *injection medicines*. *Injection medicines* are considered to be strong medicines and require doctors’ decisions, pharmacists’ review, and explanation for use by outpatients. Therefore, the economic efficiency effect for the separation of dispensing and prescribing could function normally.

The generic medicine replacement ratio has no significant partial correlation with *injection medicine* costs. The reason is considered to be the low sales in Japan of injection medicines for outpatients’ use of injections at home, such as those for insulin, growth hormones, and pain-killers for cancer. Few pharmaceutical companies sell and manufacture these medicines. However, if many pharmaceutical companies were to sell injection medicines for outpatients in the future, the generic medicine replacement ratio would have a significant partial correlation with injection medicines.

### 4.3 Medical Device Costs

*Medical device* costs had a significant partial correlation with the separation system. This is the same as the result for *injection medicine* costs. Most medical devices adopted in Japanese public insurance are used with injection medicines, like injection needles and catheters, or are considered to require doctors’ decisions, pharmacists’ review, and explanation to outpatients, because these medical devices need expert knowledge for usage. Therefore, the economic efficiency of the separation system is considered to function in this field as it does for *injection medicines*.

In Japanese regulation, generic medicine can be substituted for brand-name medicine by community pharmacists when doctors have not forbidden substitution and patients have agreed to substitution. Although there is no regulation of generic medical devices in Japan, when considering the need for patients’ agreement, patients in prefectures where the generic medicine replacement ratio is high are considered to have significant interest in the price of medical devices. As the generic medicine replacement ratio is considered to have no significant correlation with *medical device* costs, it does not influence daily medical device costs.

### 4.4 Limitations of This Study

Although we found that the daily costs of *total, internal*, and *injection medicines* had significant multiple correlations with the expansion rate and the generic medicine replacement ratio, the particular relationships denoting causes and effects remain obscure. While there are some possible reasons, they are only hypotheses and this study was unable to establish them. For a clear explanation, we have to analyze the actions of patients, doctors, and pharmacists. This remains for further research.

## 5. Conclusions

The results suggest that the separation system is effective in reducing medicine costs for *total*, *internal*, and *injection medicines* as well as *medical devices*. However, *external medicines* had no observable effect in reducing costs. It was notable that in most cases, the economic efficiency of the separation system functioned normally, although this was not the case for *external medicines*. This suggests that the cost-efficiency effect of the separation system does not function all the time.
